# Abduction Arthro-Fluoroscopy in Adhesive Capsulitis of the Shoulder

**DOI:** 10.5334/jbsr.3036

**Published:** 2023-04-06

**Authors:** Regis Sontou, Alain Nchimi

**Affiliations:** 1 CHRSM Site Sambre, BE; 2 Luxembourg Hopital Center, BE

**Keywords:** adhesive capsulitis, abduction arthro-fluoroscopy, articular pressure, scapulo-humeral block, shoulder elevation

## Abstract

This technical note describes a method of diagnosing adhesive capsulitis of the shoulder based on the real-time study of abduction movement under fluoroscopy control after opacification of the joint cavity with contrast media. This movement passively or actively shows a limitation of the abduction, a scapulohumeral block, or a weak or even an absence of rolling of the humeral head in the glenoid cavity, transforming the abduction into a shoulder elevation.

## Introduction

Adhesive capsulitis is a painful condition of the shoulder associated with restriction of passive and active movements and fibrosis of the joint capsule. The diagnosis of this condition is mostly clinical, but radiological examinations are often performed to confirm it or to rule out alternative diagnoses such as rotator cuff tendinosis or rupture, subacromial bursitis, or acromioclavicular osteoarthritis [1].

Imaging diagnosis of adhesive capsulitis lacks sensitivity and specificity. Positive but inconsistent signs of adhesive capsulitis in imaging include thickening of the coraco-humeral ligament on ultrasound, high signal intensity thickening of the joint capsule of the lower glenohumeral recess in proton density weighted with fat saturation in magnetic resonance imaging (MRI). It should be noted that the reduction of passive and active mobility of the glenohumeral joint in adhesive capsulitis is the main sign on clinical examination.

We describe the technique of abduction arthro-fluoroscopy to demonstrate the existence of the scapulo-humeral block and help establish the diagnosis of adhesive capsulitis.

## Method

In this article we describe a clinical case and the technique of abduction arthro-fluoroscopy of the shoulder. A 47-year-old woman was referred by her physician for computed tomography (CT) arthrography due to chronic pain of the right shoulder. The patient described first as a progressive onset of the shoulder pain in the previous three months, followed by a functional limitation. Pain and motion limitation were not improved by painkillers and physiotherapy. On clinical examination in a sitting position, the passive abduction movement caused pain and did not exceed 90°.

## Technique

On the pre-arthrography radiographic examination (Figure 1a), there was no periarticular calcification. The subacromial space was normal. Patient was placed in a supine position. After skin disinfection and placement of a 22G needle in the lower glenohumeral recess, 10ml of undiluted contrast media was injected.

**Figure 1a:**
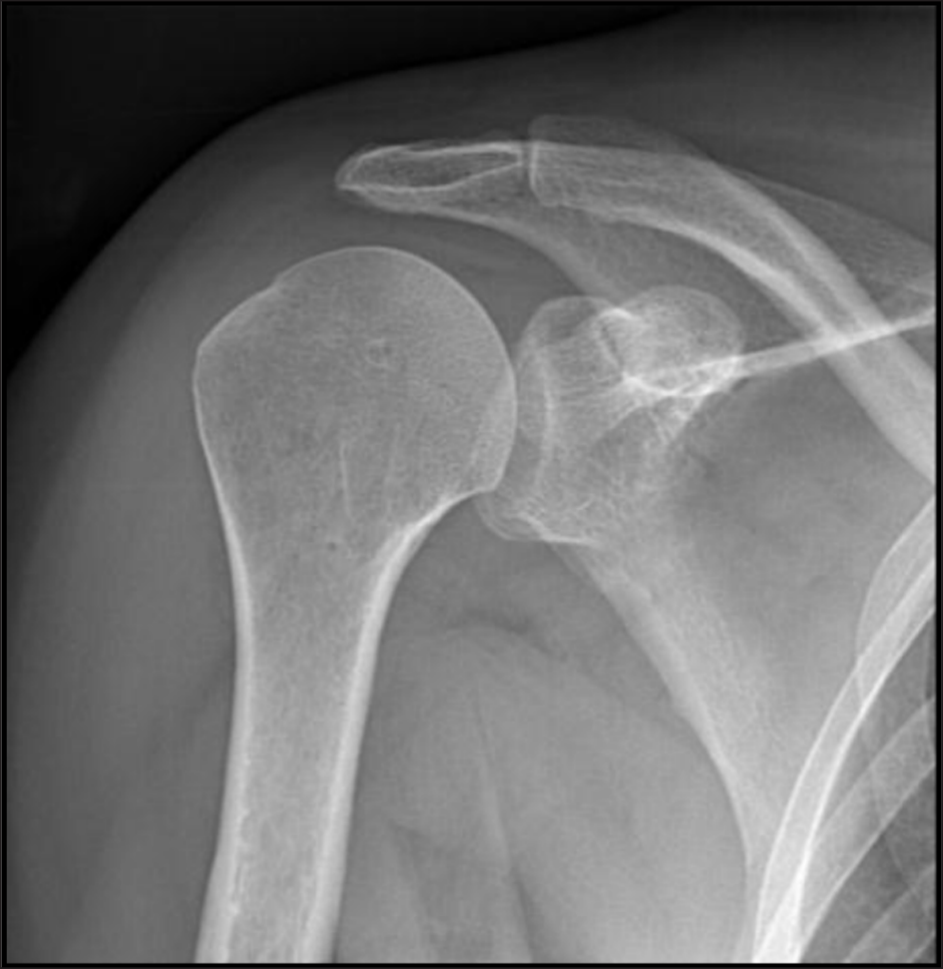
Neutral position 10°; glenohumeral and acromioclavicular joints are normal. Subacromial space is normal. No calcification of soft tissues.

The synovial relief was regular. We observed physiological opacification of the sheath of the biceps long tendon and no opacification of the subacromial space. At the end of injection, there was an appearance of an extravasation of the contrast media to the subscapularis muscle linked to increase intra-articular pressure (Figure 1b, arrow).

**Figure 1b:**
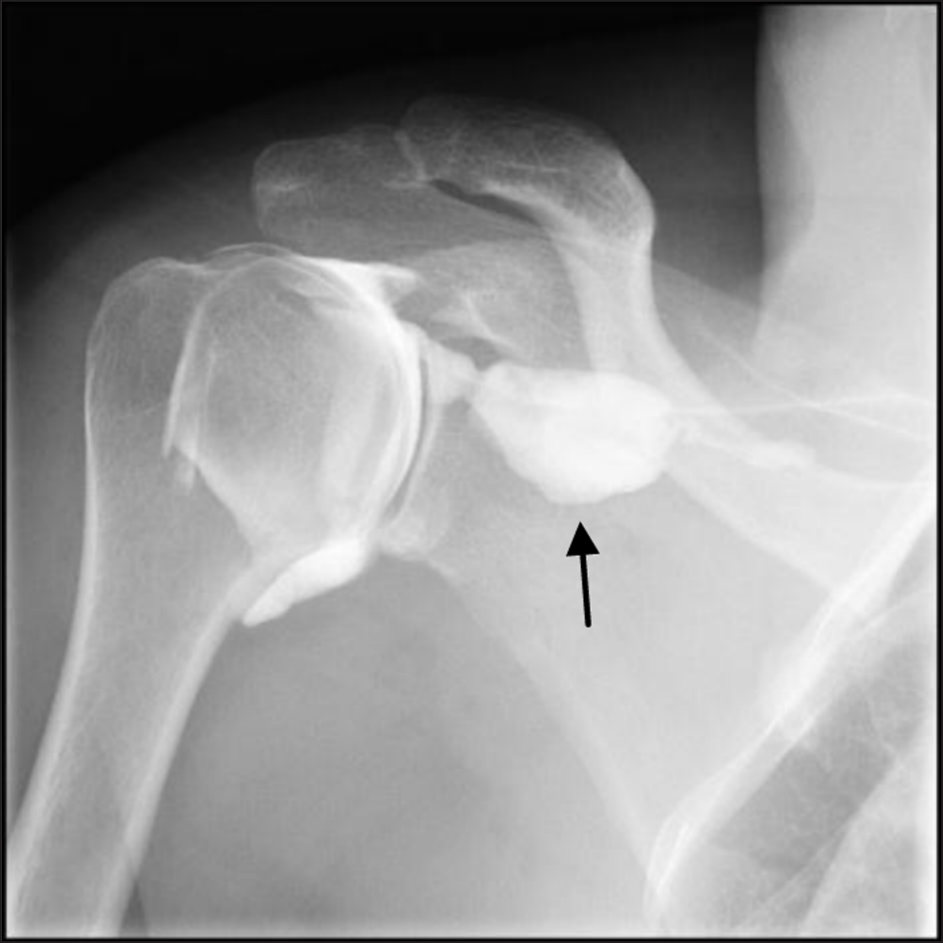
Opacification with the contrast media; the cuff is waterproof. Intra-articular pressure causes extravasation of the contrast media to the subscapularis muscle (arrow).

Prior to abduction arthro-fluoroscopy, the radiologist demonstrated to the patient the abduction by moving his arm away from the axis of the body to the maximum amplitude and bringing it back to the initial position. After instruction, the patient executed the same roundtrip arm movement under fluoroscopy. The maximal amplitude obtained was 70°–75°. A scapulohumeral block was observed during the period that extended from the spacing of the arm from the body axis to its return to the initial position. This scapula-humeral block transforms the abduction in an elevation of the scapula and humerus (Figure 1c, arrow). Rotation of the humeral head in the glenoid cavity was almost non-existent (Figure 1d, star). The increased articular pressure leads to increase extravasation of the contrast media (Figure 1d, head arrow). Passive abduction performed by the radiologist identically reproduced the scapula-humeral block.

**Figure 1c:**
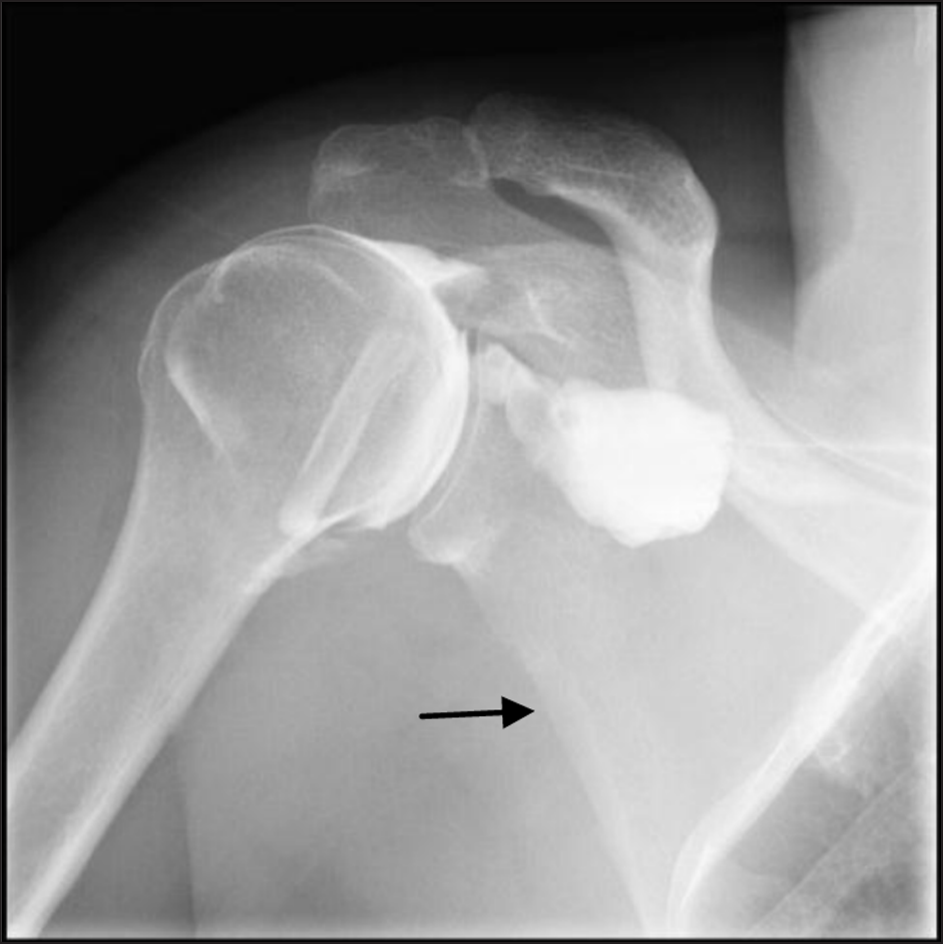
Abduction 30° with internal rotation; there is a beginning of ascent of the scapulohumeral block (arrow).

**Figure 1d:**
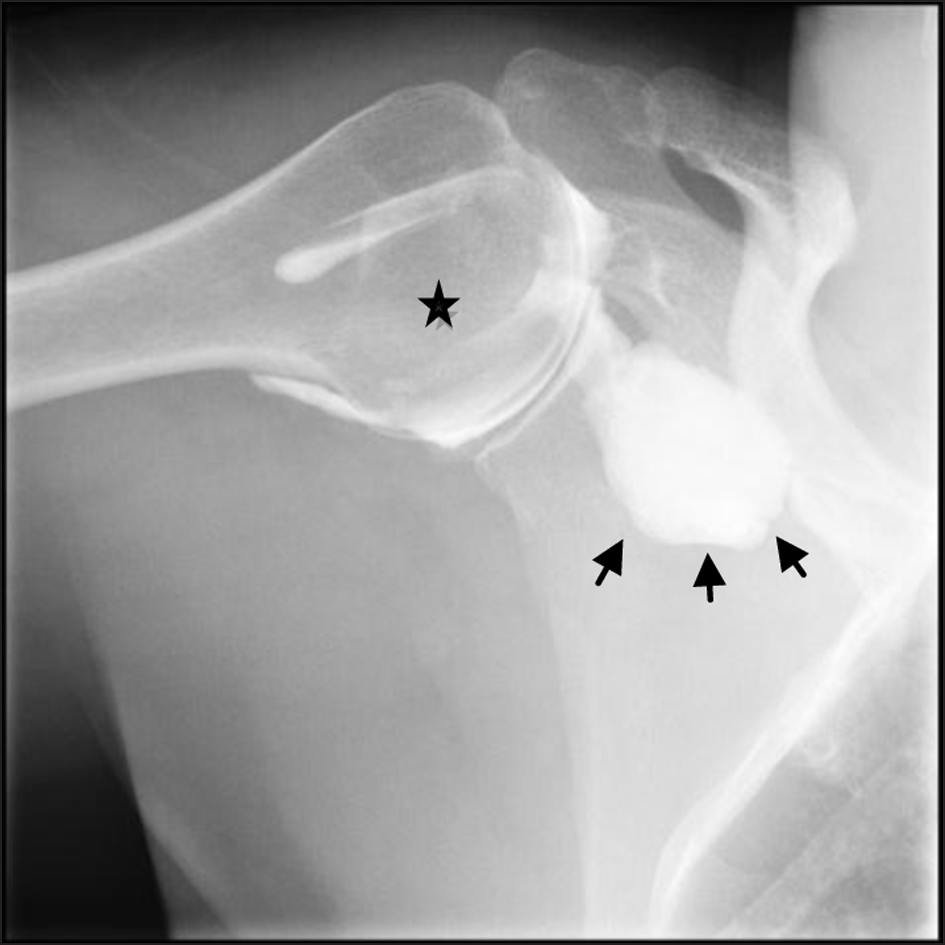
Maximum abduction obtained at 70–75°; the glenohumeral couple achieves a cranial ascent and weak roll of the humeral head in the glenoid cavity (star). Increase of the extravasation of the contrast media (head arrow).

CT performed after arthrography showed normal findings. The diagnosis of adhesive capsulitis is retained in absence of damage to the rotator cuff. The patient underwent thirty sessions of physiotherapy and two intra-articular infiltrations of corticosteroid. After three months, the recovery of range of motion was almost complete.

## Discussion

Adhesive capsulitis is clinically characterized by a blockage of passive and active movements (especially abduction) of the shoulder. The normal amplitude of this movement is around 180°. During abduction, the humeral head rolls in on the glenoid cavity and the scapula is slightly mobile.

Abduction arthro-fluoroscopy technique that we described is a dynamic technique that allows for real-time visualization of the abduction of the shoulder and the limitation of the amplitude below 90°. The humerus and scapula form a block and transform the abduction in a shoulder elevation. The contrast media provides information on the cuff. This examination requires no anesthesia. Although only subsequent studies will determine its value, we believe that, based on the benefits described above, this diagnostic approach is the most complete when a radiological examination is required in the context of suspected adhesive capsulitis of the shoulder.

The treatment for adhesive capsulitis of the shoulder is uncertain. Physiotherapy and non-steroidal anti-inflammatory drugs are offered as a first line to try to recover mobility and manage pain. Intra-articular infiltrations of corticosteroid are proposed as a second line. If pain and functional restriction persist after six months, the surgical option should be considered. It offers either manipulation under anesthesia or capsular release under arthroscopy [2].

## Conclusion

Abduction arthro-fluoroscopy of the shoulder is a specific technique for real-time observation of abduction movement. The patient discussed here showed a scapulohumeral block, shoulder elevation, and extravasation of the contrast media in the scapularis muscle.

## References

[1] Ewald A. Adhesive capsulitis: a review. Am Fam Physician. 2011; 83(4): 417–22. 21322517

[2] Ramirez J. Adhesive capsulitis: diagnosis and management. Am Fam Physician. 2019; 99(5): 297–300. 30811157

